# GABAergic Circuit Development and Its Implication for CNS Disorders

**DOI:** 10.1155/2011/623705

**Published:** 2011-10-17

**Authors:** Graziella Di Cristo, Tommaso Pizzorusso, Laura Cancedda, Evelyne Sernagor

**Affiliations:** ^1^CHU Sainte-Justine Research Center and Department of Pediatrics, Université de Montréal, Montreal, QC, Canada H3T1C5; ^2^Department of Psychology, University of Florence, 50121 Florence, Italy; ^3^Institute of Neuroscience CNR, 56100 Pisa, Italy; ^4^Italian Institute of Technology, 16163 Genova, Italy; ^5^Institute of Neuroscience, Medical School, Newcastle University, Newcastle upon Tyne NE2 4HH, UK

The function of the cerebral cortex requires the coordinated action of two major neuronal subtypes, the glutamatergic projection neurons and the GABAergic interneurons. Although, in terms of numbers, GABAergic interneurons represent a minor cell population compared to glutamatergic neurons in the neocortex, they play an important role in modulating network dynamics of neocortical circuits. Indeed, GABAergic interneurons have been shown to control neuronal excitability and integration, and they have been implicated in the generation of temporal synchrony and oscillatory behavior among networks of pyramidal neurons. Such oscillations within and across neural systems are believed to serve various complex functions, such as perception, movement initiation, and memory. Recently, the development of GABAergic inhibition has been shown to be a key determinant for critical period plasticity of cortical circuits. Critical periods represent heightened epochs of brain plasticity, during which experience can produce permanent, large-scale changes in neuronal circuits. Experience-dependent refinement of neural circuits has been described in many regions within the CNS, suggesting it is a fundamental mechanism for normal vertebrate CNS development. By regulating the onset and closure of critical periods, GABAergic interneurons may influence how experience shapes brain wiring during early life and adolescence. 

Considering the multifaceted role played by GABAergic cells in the development, function, and plasticity of neural circuits, it is not surprising that alterations in the development of GABAergic circuits per se have been implicated in various neurodevelopmental and psychiatric disorders such as schizophrenia, autism, and epilepsy. However, how modification of GABAergic circuit development contributes to specific pathologies is largely unknown. Furthermore, GABA mimetic drugs, such as benzodiazepines and certain antiepileptic drugs, are widely used in clinical practice, but whether and to what extent these drugs cause deleterious effect on the developing brain is still not clear. A better comprehension of the mechanisms underlying the development and plasticity of GABAergic interneurons will likely indicate which cellular substrates might be affected in neurodevelopmental disorders. At the same time, identifying the genetics variants implicated in these disorders may generate major new insights into the normal and pathological function of GABAergic circuits.

Our understanding of GABAergic interneurons function is challenged by their startling heterogeneity; indeed, different subtypes of interneurons display distinct morphology, physiological properties, connectivity patterns, and biochemical constituents. Recent technical advances have significantly accelerated progress in this field. In particular, the development of genetic strategies based on interneuron cell type-specific promoters and fluorescent protein reporters has allowed efficient high-resolution labelling of specific GABAergic interneuron classes in intact or semi-intact tissues, such as organotypic brain cultures.

Contributions to this special issue of provide an overview of recent discoveries in the field of GABAergic circuit development and related brain disorders. The genetic program for the construction of cortical GABAergic network is initiated early during brain development, and it orchestrates cell type specification, migration, and some aspects of synaptic connectivity. On the other hand, the establishment of mature patterns of GABAergic innervation and inhibitory transmission is not achieved until adolescence and is profoundly influenced by neuronal activity and experience. E. Rossignol describes the tightly controlled genetic cascades that determine the great diversity of cortical GABAergic interneurons and how dysfunctions in genes important for their generation, specification, and maturation might contribute to various neurodevelopmental disorders. B. Chattopadhyaya describes the molecular mechanisms underlying the activity-dependent maturation of GABAergic innervation in the postnatal brain.

 Several articles in the special issue have investigated the evidence linking dysfunction in GABAergic signaling and plasticity to specific neurodevelopment disorders, such as autism (R. Pizzarelli and E. Cherubini, J. LeBlanc and M. Fagiolini, L. Baroncelli et al.), schizophrenia (G. Gonzales-Burgos et al.), and epilepsy (Griggs and Galanopoulou). The developmental role of GABAergic circuits is not limited either to the brain or to the developmental phase. A. E. Allain et al. discuss the role of GABA and GABAergic receptors in motoneuron development and in immature hypoglossal motoneurons of the spastic mouse, a model of human hyperekplexic syndrome. B. Imbrosci and T. Mittman describe the response of the GABAergic system to cortical injuries in the adult and how this response could be manipulated to help the functional recovery of patients.

In the last decades, cell-based therapies using GABAergic neuronal grafts have emerged as a promising treatment, since they may restore the lost equilibrium by cellular replacement of the missing/altered inhibitory neurons or modulating the hyperactive excitatory system. Advances in this field are reviewed by V. Broccoli and M. Dolado.

 It is becoming increasingly clear that the strength of GABAergic synaptic transmission is dynamic. R. Wright et al. review some of the sophisticated ways in which GABA-A receptor driving force can vary within neuronal circuits. P. Méndez and A. Bacci discuss the plasticity and modulation of adult cortical and hippocampal GABAergic synaptic transmission, while P. Wenner describes new insight into the mechanisms of GABAergic homeostatis in developing motor networks. Finally, A. Ludwig et al. provide evidence that the trophin nurturin is implicated in the developmental regulation of the cotransporter KCC2, a key molecular player in the establishment of the chloride-gradient, which in turn regulates the strength of GABAergic transmission.

We hope that this special issue will serve to emphasize the new technical and conceptual advances in the field of GABAergic circuits development and to highlight the importance of this network for neurological disorders.

*Graziella Di Cristo* *Graziella Di Cristo* *Tommaso Pizzorusso* *Tommaso Pizzorusso* *Laura Cancedda* *Laura Cancedda* *Evelyne Sernagor* *Evelyne Sernagor*

